# Feasibility of AI-Denoised Ultra-Low-Dose CT for Detection of Acute Pelvic and Hip Fractures: A Pilot Multi-Reader Study

**DOI:** 10.3390/diagnostics16172862

**Published:** 2026-09-06

**Authors:** Daniel Nguyen, Tina Shiang, Connie Ge, George Watts, Christopher Sereni, David Radcliffe, Hemang Kotecha, Gabriela Santos Nunez, Young H. Kim

**Affiliations:** 1 Department of Medicine, University of Massachusetts Chan Medical School, Worcester, 01655 MA, USA; 2 Department of Radiology, University of Massachusetts Chan Medical School, Worcester, 01655 MA, USA; 3 Department of Radiology, Massachusetts General Hospital, Boston, 02114 MA, USA; 4 Department of Radiology, Vanderbilt University, Nashville, 37235 TN, USA; 5 VRad, Minneapolis, 55435 MN, USA

**Keywords:** diagnostic imaging, deep learning, radiology, artificial intelligence, radiation dosage, X-ray CT, low dose, denoised

## Abstract

**Objective:** To evaluate the feasibility of simulated ultra-low-dose CT (ULD-CT) enhanced with deep learning denoising (DLD) for detecting pelvic and hip fractures. **Method:** This Institutional Review Board-approved retrospective study was performed at a multisite, single academic institution between October 2020 and May 2021, including 30 full-dose CT (CTfull) exams of acute pelvic and hip trauma (10 normal, 10 “easy,” 10 “hard” cases by fracture conspicuity). Simulated low-dose CT images at 10% (CT10sim) and 5% (CT5sim) radiation dose were generated using a noise model-based algorithm and reconstructed from full-dose CT datasets. A commercial deep learning denoising algorithm enhanced these images, resulting in denoised low-dose CTs (CT10dld and CT5dld) for evaluation. The 90 resulting CT cases were reviewed independently for diagnostic accuracy, confidence, sufficiency, and imaging quality. **Result:** Diagnostic accuracy was similar across CT modalities overall (CTfull 80.8% [72.9, 86.9]; CT10dld 78.3% [70.1, 84.8]; CT5dld 77.5% [69.2, 84.1]) and within the easy and normal strata; in hard cases, accuracy was numerically lower for CT10dld (45.0%) and CT5dld (42.5%) than for CTfull (57.5%). Diagnostic sufficiency was similarly high across modalities (CTfull 91.7%, CT10dld 87.5%, CT5dld 88.3%). CT full was rated highest in perceived image quality across all strata (mean 4.21 ± 0.83 overall vs. 3.47 ± 0.97 for CT10dld and 3.23 ± 0.98 for CT5dld) and in diagnostic confidence (mean 4.61 ± 0.63 vs. 4.45 ± 0.71 and 4.41 ± 0.72, respectively), though neither translated into a corresponding drop in diagnostic accuracy overall. Inter-reader agreement was highest between MSK radiologists, reaching substantial agreement on CT10dld (κ = 0.760) and CT5dld (κ = 0.672). **Conclusions:** DLD-enhanced ULD-CT showed diagnostic accuracy and sufficiency similar to full-dose CT for pelvic and hip fracture detection, at a fraction of the radiation dose, though full-dose CT was rated higher in perceived image quality and diagnostic confidence and outperformed denoised low-dose CT numerically in hard cases. Full-dose CT remains the preferred modality for complex and high-energy trauma, while denoised low-dose CT modalities represent a promising option for straightforward presentations where radiation minimization is a priority.

## 1. Background

Pelvic and hip fractures are significant injuries commonly encountered in the emergency department (ED), either in the context of high-energy trauma such as motor vehicle collisions or low-energy incidents like falls in older adults [1,2]. Accurate and timely diagnosis is crucial to prevent complications such as chronic pain, immobility, or even death. Misdiagnosis or delayed diagnosis of hip fractures can also lead to medicolegal issues, including lawsuits [3,4,5]. Imaging plays a central role in the evaluation and management of these injuries. In current clinical practice, pelvic radiographs (XR) are typically the first-line imaging modality due to their accessibility, speed, and low cost. However, their sensitivity in detecting subtle or complex fractures, such as occult fractures or those in challenging anatomical regions, is limited. When radiographs are inconclusive, computed tomography (CT) is often utilized for further characterization. CT offers superior diagnostic accuracy and three-dimensional imaging capability, making it invaluable for identifying fractures missed by XR, particularly in anatomic areas that are diagnostically challenging, like the acetabulum and sacrum.

Despite the diagnostic advantages of CT for fracture detection, the routine use of CT raises concerns about cumulative radiation exposure for the patient. The risk associated with radiation exposures is well documented in the literature, especially the albeit low, but notable, increased lifetime risk of malignancy where one study showed a lifetime attributable risk of 1.7% above baseline cancer risk [6]. Further, trends have revealed that the number of CT scans performed continues to rise, increasing the cumulative radiation exposure for each patient [7,8,9,10]. These considerations ultimately drive the need for radiation dose reduction while maintaining diagnostic accuracy in the trauma setting. However, since reducing the radiation dose inevitably affects CT image quality, there continues to be active research in finding strategies that balance radiation risk and diagnostic performance [11,12].

Deep learning-based denoising software (DLD) have been developed to address the trade-off between dose reduction and image quality [13,14]. These algorithms utilize complex neural networks to denoise and enhance the diagnostic quality of images acquired at lower radiation doses such as ultra-low dose CT (ULD-CT) [15]. Several studies have evaluated the use of deep learning to improve the image quality in CT scans, which have been shown to provide similar imaging quality between simulated ULD-CT compared to standard, or full-dose, CT [16,17,18]. The advantages of ULD-CT over XR extend beyond radiation safety, as three-dimensional evaluation with better spatial resolution often yields more accurate assessments. Thus, when these algorithms are applied to full-dose CT, they hold significant promise in helping the research community understand the value of ULD-CT as an alternative to radiographs in first-line imaging.

While the benefits of ULD-CT are compelling for widespread clinical adoption, further research is needed to determine its reliability in detecting pelvic and hip fractures compared to standard-dose CT. Another point to consider is the potential variability in diagnostic accuracy among radiologists with different subspecialty training, such as emergency (ED) radiologists and musculoskeletal (MSK) radiologists. Third, complexity of the fracture and the patient’s baseline bone mineralization may impact the diagnostic performance of ULD-CT. While ULD-CT may be determined as a reliable imaging modality for fractures that are easily identified on imaging, its performance in detecting more challenging fractures may be less reliable. These key considerations are crucial in determining the value of incorporating ULD-CT in the ED trauma setting.

To our knowledge, no prior study has evaluated a commercial deep learning denoising algorithm applied to simulated ULD-CT specifically for detection of acute pelvic and hip fractures, using a multi-reader, multi-specialty design that separates cases by fracture conspicuity. This gap matters because pelvic and hip trauma imaging carries distinct diagnostic challenges: subtle nondisplaced fractures and a patient population (often older adults) in whom cumulative radiation exposure and time-sensitive diagnosis are both clinically important. In this pilot study, we address this gap by evaluating the feasibility of DLD-enhanced ULD-CT as a safer imaging modality in specific trauma triage scenarios. By applying a deep learning-based denoising algorithm to simulated ULD-CT images, we assess diagnostic accuracy and inter-reader reliability among ED and MSK radiologists for detecting pelvic and hip fractures in acute trauma. As a feasibility pilot, this study is intended to generate hypotheses and inform the design of an adequately powered prospective trial, rather than to establish clinical equivalence or guide an immediate change in imaging protocol.

## 2. Methods

Our institutional review board approved this Health Insurance Portability and Accountability Act-compliant study. The requirement for informed consent of patients was waived.

### 2.1. Inclusion/Exclusion Criteria

**Figure 1:**
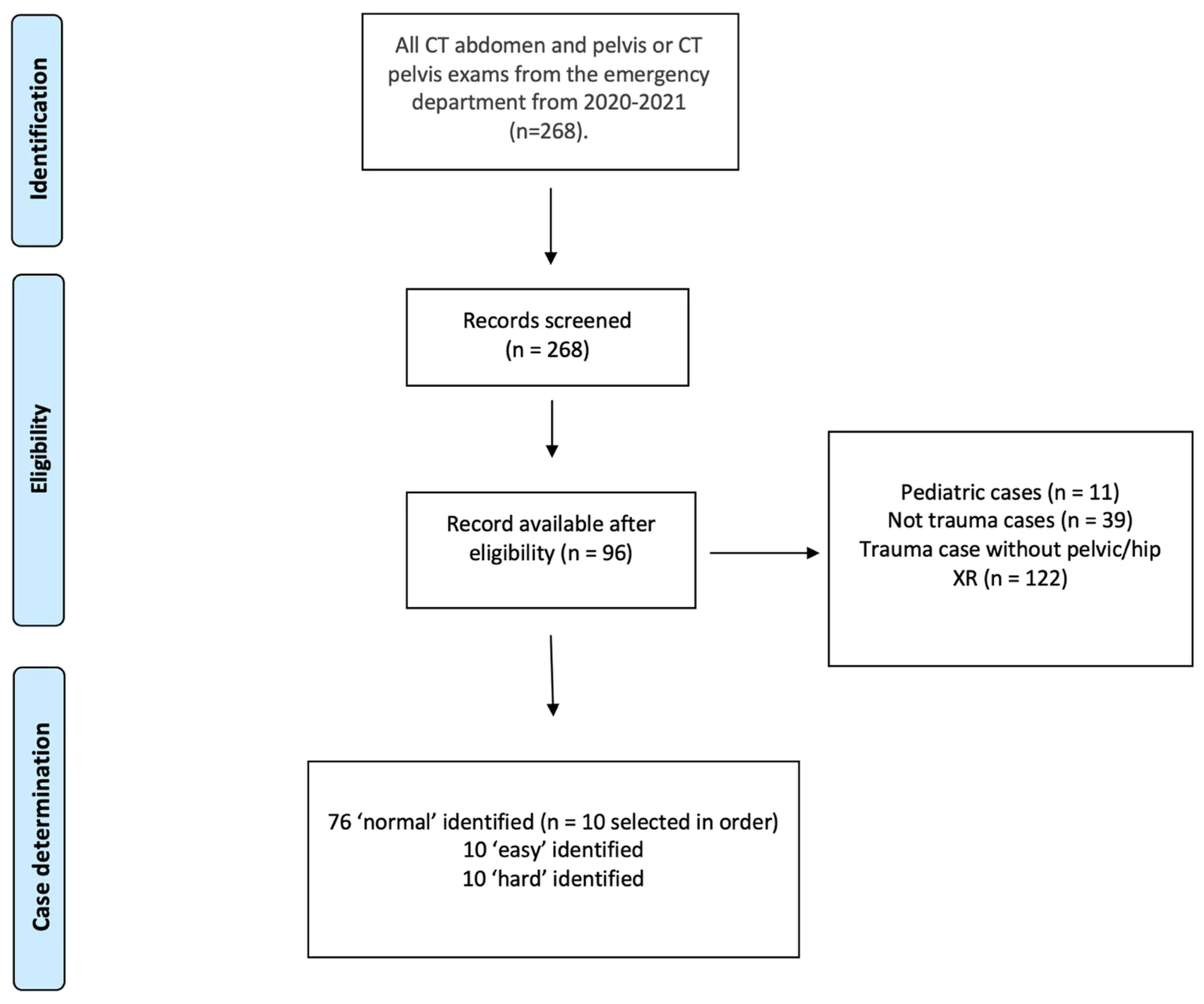
Flow chart of exclusion and inclusion criteria for the study. Cases with substantial image-quality limitations (e.g., marked demineralization, hardware artifact, large body habitus) are included within the exclusion categories above rather than tracked separately, as these characteristics typically co-occurred with other exclusion criteria (see Section 2).

A retrospective cohort study of 30 adult patients was performed at a multisite, single academic institution between October 2020 and May 2021. A total of 30 cases were selected after consultation with a statistician. Inclusion criteria include all adult (>18 years old) trauma patients who presented to our emergency department with suspected pelvic or hip fracture. Each patient was required to have both radiographs (pelvic or hip radiographs) as well as a concurrent or later CT during the initial trauma evaluation (including CT abdomen pelvis, CT pelvis, CT reconstruction pelvis). Both contrast-enhanced and non-contrast CT exams were eligible for inclusion, reflecting a realistic case-mix for evaluation of acute pelvic and hip trauma in the ED setting. Exclusion criteria, applied prior to case review, included: pediatric patients (*n* = 11); imaging obtained for post-operative evaluation or for pelvic/hip pain from non-traumatic causes such as systemic disease, tumor, or infection (*n* = 39, “not trauma”); and trauma cases without a corresponding radiograph (*n* = 122) (Figure 1). As part of this same a priori screening, cases were also required to be of adequate diagnostic image quality; patients with substantial osseous demineralization, hardware-related artifact, or large body habitus typically did not meet inclusion criteria for other reasons (e.g., non-traumatic indication) and so are captured within the exclusion categories above rather than tracked as a separate category. Images were retrieved retrospectively from the clinical data repository from May 2021 until the adequate number of cases for normal, “easy”, and “hard” cases (further described below) were obtained (further described below), and subsequently de-identified and anonymized. Figure 1 demonstrates the inclusion and exclusion flow chart.

### 2.2. Case Selection

CT abdomen and pelvis trauma exams from the emergency department were reviewed consecutively in reverse chronological order until 30 CTs that met the inclusion criteria were identified. Of these 30 CT’s, 23/30 (76.7%) were contrast-enhanced and 7/30 (23.3%) were non-contrast-enhanced. Cases include 10 normal, 10 with “easy” fracture findings, and 10 with “hard” fracture findings. The ground truth was determined by a board-certified musculoskeletal radiologist (G.W.) and PGY-5 radiology resident (T.S.) for case difficulty level and imaging quality. The two adjudicators reviewed each case independently and reached consensus on case difficulty by discussion; ground truth diagnosis was based on CT review and the finalized radiology report, without additional follow-up imaging or clinical history. For the purposes of case stratification, fractures were categorized as “easy” or “hard” based on radiographic conspicuity and anatomical complexity. All cases were limited to hip and pelvic fractures, and fractures involving other anatomic regions (e.g., lumbar spine) were explicitly excluded. “Easy” fractures were defined as fractures that were clearly visible on both radiographs and CT with minimal diagnostic ambiguity, such as displaced femoral neck fractures. In contrast, “hard” fractures were defined as fractures that are subtle or radiographically occult on radiographs, often due to being nondisplaced or minimal displaced, comminuted, or involving anatomically complex regions with superimposition of osseous structures and are more definitively characterized on CT. Examples of “hard” fractures included acetabular fractures, sacral or coccygeal fractures, and minimally displaced or nondisplaced pubic rami fractures. While the majority of fracture cases involved a single fracture site, a subset of cases included multiple fracture sites within the pelvis and/or hip. During reader training prior to the image interpretation sessions, radiologists were explicitly informed that cases could contain one or more fractures and that the task was to identify all fracture(s) present, rather than assuming a single-fracture scenario. Cases were de-identified and anonymized before being provided to the vendor for image processing through the deep learning denoising algorithm.

### 2.3. Reduced-Dose Simulation and Denoising Algorithm

To generate simulated ultra-low-dose CT images, we used the previously described algorithm which creates simulated reduced dose images using DICOM CT images [19,20]. Briefly, the algorithm creates synthetic sinograms with artificially reduced x-ray photon flux, adds object-attenuation-dependent and system noise, and reconstructs the resulting reduced-dose images. The noise-insertion algorithm used to generate CT10sim and CT5sim models quantum-limited (photon-starved) noise rather than simple image-domain noise addition by adding synthetic noise at the sinogram level prior to reconstruction. At 5% of standard dose, the simulated photon flux approaches levels at which quantum mottle becomes visually dominant and can approach a quantum starvation regime, in which the noise floor limits the visibility of low-contrast structures independent of any subsequent processing. This is the noise condition the denoising algorithm is applied to counteract. Using this method, low-dose CT images with relative dose levels of 10% (CT10sim) and 5% (CT5sim) were computed from the original full dose CT dataset. Because the denoising method is proprietary, its source code and complete internal processing parameters were not available to the investigators, which limits independent replication and technical assessment of the algorithm.

### 2.4. Deep Learning Denoising Model

**Figure 2:**
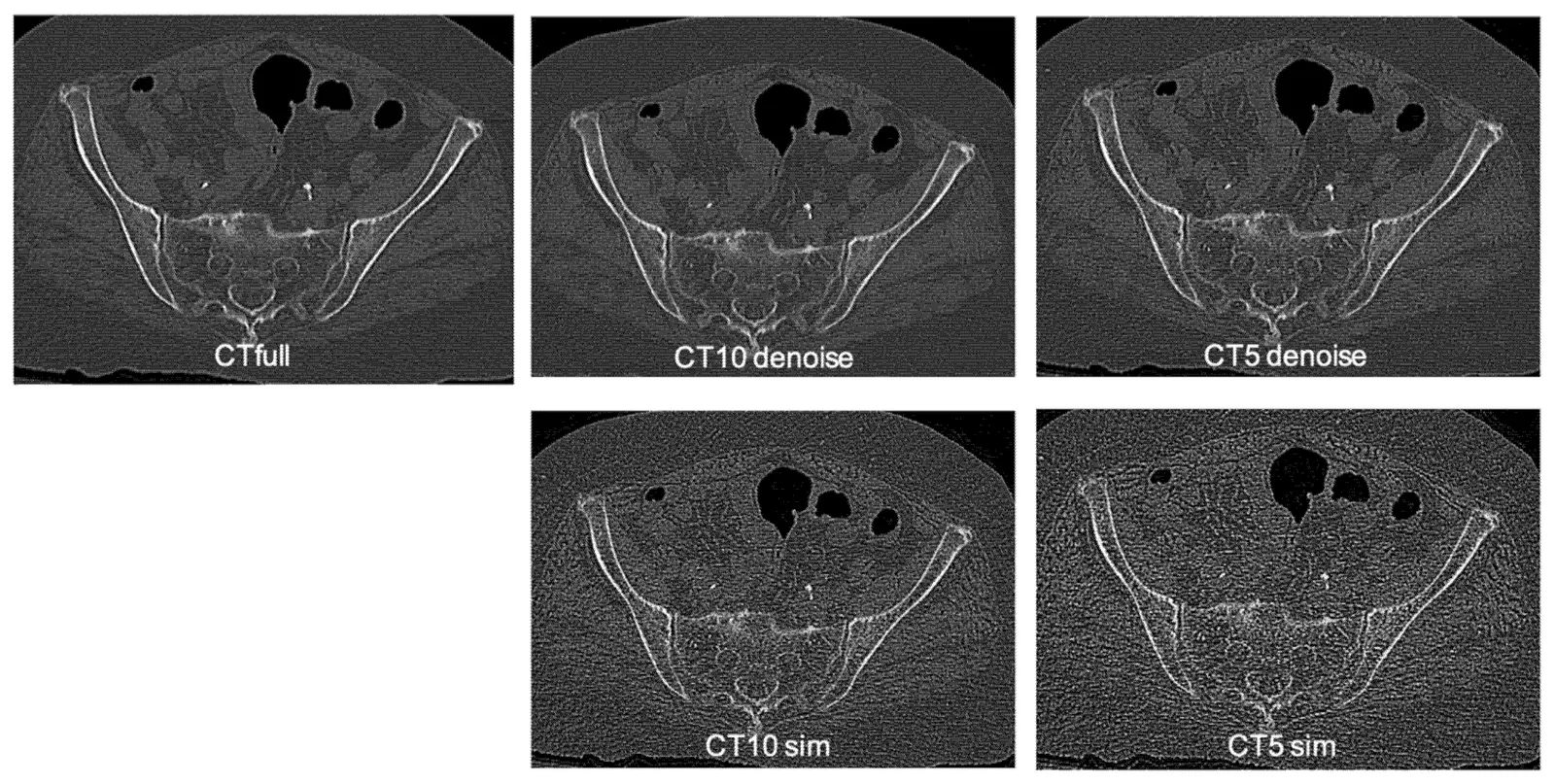
Representative images from a “normal” case of the pelvis without acute fracture. Radiation doses of CTfull is 26.12 mSv, CT10sim is 2.61 mSv, CT5sim is 1.31 mSv. CTfull = standard dose CT. CT10 denoise (dld) = 10% radiation of the standard CT after denoising algorithm. CT5 denoise (dld) = 5% radiation dose of the standard CT after denoising algorithm. CT10sim = simulated image at 10% radiation of the standard CT before denoising. CT5sim = simulated image at 5% radiation of the standard CT before denoising.

**Figure 3:**
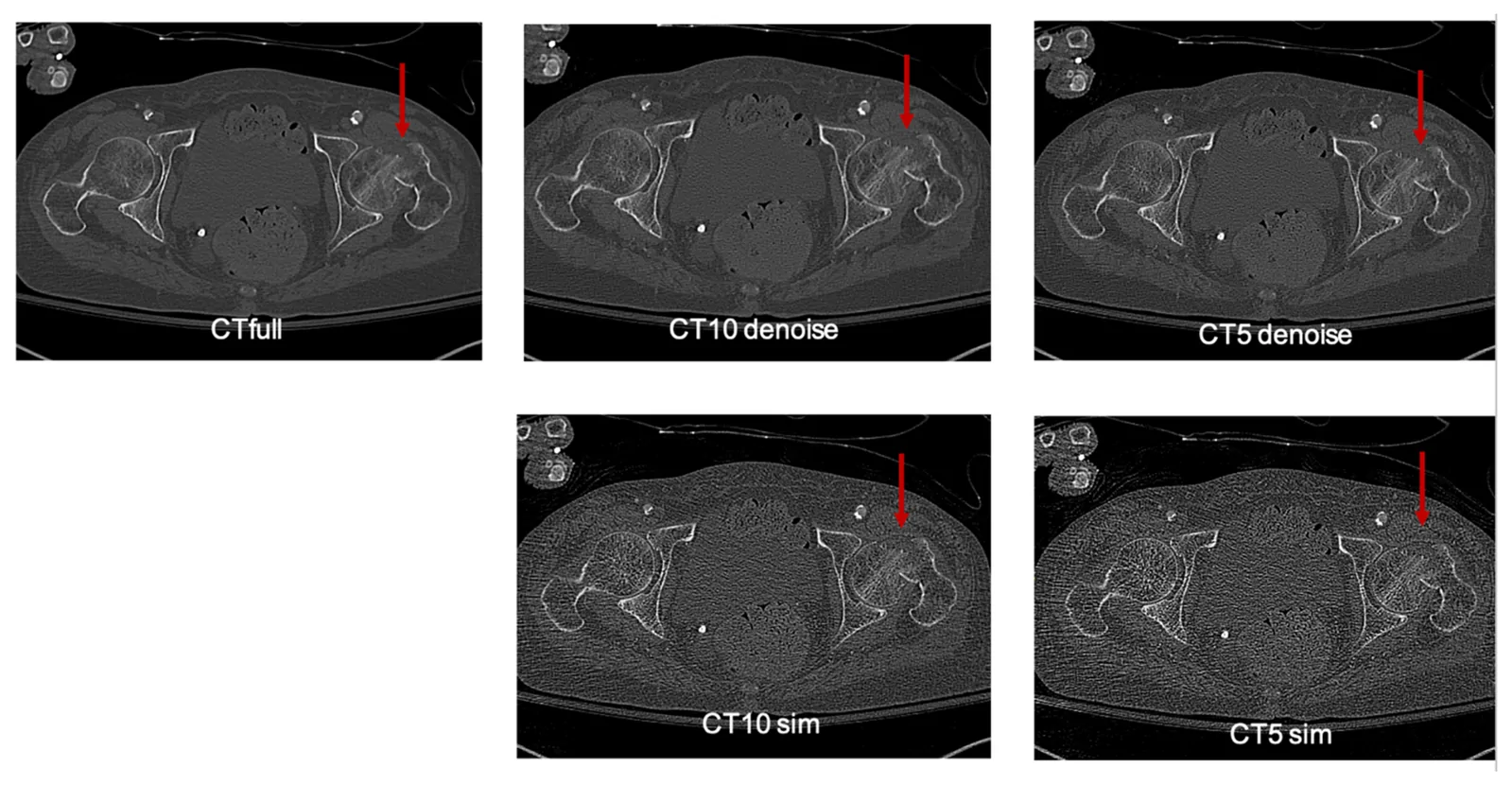
Representative images from an “easy” case demonstrating acute displaced and angulated left femoral neck fracture (red arrow). Radiation doses of CTfull is 17.09 mSv, CT10sim is 1.71 mSv, CT5sim is 0.85 mSv. CTfull = standard dose CT. CT10 denoise (dld) = 10% radiation of the standard CT after denoising algorithm. CT5 denoise (dld) = 5% radiation dose of the standard CT after denoising algorithm. CT10sim = simulated image at 10% radiation of the standard CT before denoising. CT5sim = simulated image at 5% radiation of the standard CT before denoising.

**Figure 4:**
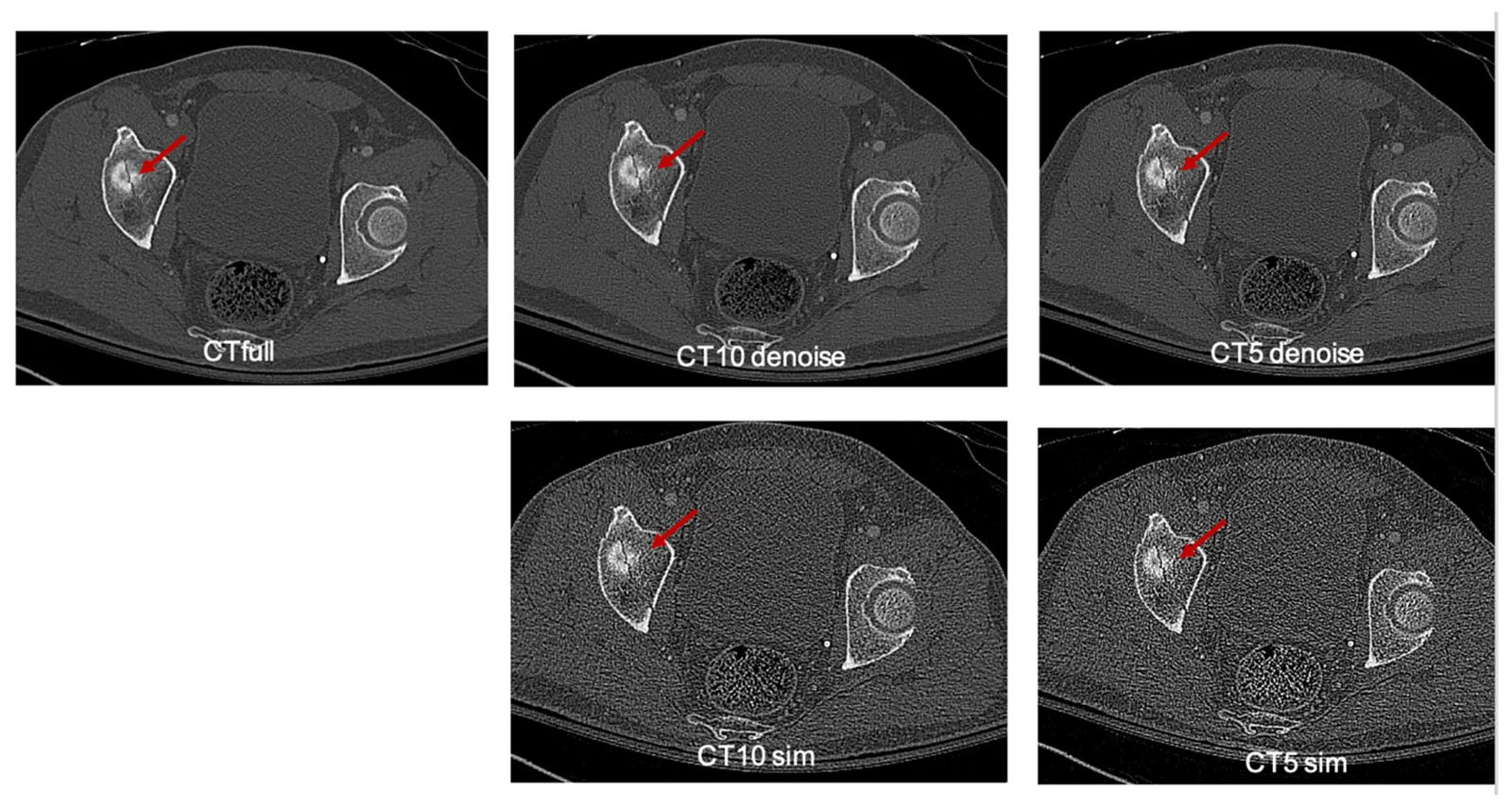
Representative images from a “hard” case demonstrating acute nondisplaced fracture of the right acetabulum (red arrow). Radiation doses of CTfull is 19.77 mSv, CT10sim is 1.98 mSv, CT5sim is 0.99 mSv. CTfull = standard dose CT. CT10 denoise (dld) = 10% radiation of the standard CT after denoising algorithm. CT5 denoise (dld) = 5% radiation dose of the standard CT after denoising algorithm. CT10sim = simulated image at 10% radiation of the standard CT before denoising. CT5sim = simulated image at 5% radiation of the standard CT before denoising.

Subsequently, a commercial deep learning CT denoising algorithm (ClariCT.AI, Version 1.0, ClariPi, Seoul, South Korea) was employed to enhance the quality of these simulated low-dose images [21]. The deep learning-based denoising (DLD) software utilized in this study is based on a U-Net Convolutional Neural Network (CNN). The model was trained using a supervised learning framework designed to map noise-added CT images to their corresponding standard-dose counterparts. To ensure vendor-agnostic capability, the training dataset comprised over 1 million images from 24 different scanner models across four major manufacturers (GE, Siemens, Philips, and Canon), encompassing 2100 combinations of scan parameters [22]. The performance of DLD has been previously evaluated within several clinical studies [23,24,25]. This process resulted in a total of 90 imaging exams, comprising 30 CTfull, 30 CT10dld, and 30 CT5dld, (dld = denoised low dose). All cases were subsequently randomized for analysis. Example images of each category (“normal”, “easy”, and “hard”) are provided in Figure 2, Figure 3 and Figure 4. These figures show the original image (denoted as CTfull), the simulated image at different radiation dose levels without denoising (CT10sim, and CT5sim), and the denoised image (CT10dld, and CT5dld).

### 2.5. Readers

The cases were independently evaluated by four fellowship-trained attending radiologists, including two musculoskeletal radiologists (C.S. and D.R., with 5 years and 1 year of post-training experience, respectively) and two emergency radiologists (H.K. and G.S., with 4 years and 3 years of post-training experience, respectively), in three different reading sessions of 30 cases (10 “normal”, 10 “easy”, and 10 “hard) each with 2-week washout period between reading sessions. The total number of images read per radiologist was 90. Prior to the reading sessions, a training session was held to familiarize the readers with the structure of the reading sessions, survey questions, PACS, and answer any questions to help standardize the reading sessions. Readers were presented with randomized cases in mixed batches of CT without context of the relationships between the radiographs, full dose, and reduced dose CTs (CTdld). To mitigate bias, no clinical information or associated prior images were provided. Readers were asked to provide a diagnosis from the imaging (Question 1), rate their confidence in the diagnosis (Question 2), and assess the diagnostic sufficiency (Question 3) and quality of the imaging (Question 4) in the survey (Figure S1). Responses for Question 1 were reviewed and graded as either correct or incorrect (including misdiagnosis, incomplete diagnosis, or overcall). Incomplete diagnosis is defined as reporting some, but not all, of the pertinent imaging findings. All readers (100%) responded to the survey. Ground truth was established by CT review, consensus between musculoskeletal radiologist and radiology trainee, and the finalized radiology report rather than a more sensitive reference standard such as bone scintigraphy or MRI, so we cannot exclude that the single discordant “normal” case represents an occult fracture missed by our reference standard rather than a false-positive read.

### 2.6. Radiation Dose Calculation and Conversion

Comparison of radiation doses between CT was performed with guidance from a medical physicist (B.L.). Radiation doses for CT are reported in the dosimetry report using dose-length product (DLP), and estimated effective radiation dose (mSv) was calculated by multiplying the conversion factor 0.015 mSv/(Gy·cm) and the DLP [26,27]. The DLP-derived effective dose is a population-level approximation rather than a patient-specific organ- or absorbed-dose estimate, and it may vary with the anatomical irradiation field, scan range, patient size and age, scanner characteristics, and the conversion-coefficient source used. Further, because CT5sim and CT10sim were simulated images, the mSv reported for these figures are nominal simulated doses.

### 2.7. Statistical Analysis

All statistical analyses were performed with Python (v3.12). Diagnostic responses were binarized as correct (1) or incorrect (0), where any response other than “correct” was classified as “incorrect” (miss, incomplete, or overcall). Confidence intervals for accuracy proportions of 95% were computed using the Wilson score interval. Diagnostic confidence (Question 2) and perceived image quality (Question 4), both collected on 1–5 Likert scales, were analyzed using descriptive statistics including median, interquartile range (IQR), and standard deviation. Diagnostic sufficiency (Question 3) was summarized descriptively as the proportion of reads rated “sufficient” per modality, with Wilson score 95% confidence intervals. Inter-reader agreement on diagnostic accuracy (Question 1) was assessed using Cohen’s kappa, calculated separately for each CT modality, and interpreted according to the thresholds of Landis and Koch [28].

## 3. Results

In this cohort of 30 patients, the mean age was 59.3 years (median: 59.0, IQR: 44.0–74.0), and the cohort consisted of 16/30 (53.3%) male and 14/30 (46.7%) female.

Across all four radiologists (ED1, ED2, MSK1, and MSK2) for thirty cases, diagnostic accuracy was high across all CT modalities. CTfull achieved the highest accuracy (97/120 reads, 80.8% [95% CI: 72.9–86.9]), followed by CT10dld (94/120, 78.3% [70.1–84.8]) and CT5dld (93/120, 77.5% [69.2–84.1]) (Table 1). Accuracy, however, varied markedly by case difficulty. For easy cases, all three modalities performed similarly and at high levels (CTfull 87.5%, CT10dld 92.5%, CT5dld 92.5%). Normal cases were nearly perfectly characterized across the board (97.5% for all three modalities). However, this improvement is likely due to sampling variation rather than true dose-dependent improvement. Hard cases showed the greatest divergence, with CTfull achieving the highest accuracy (23/40, 57.5% [42.2–71.5]) compared to CT10dld (18/40, 45.0% [30.7–60.2]) and CT5dld (17/40, 42.5% [28.5–57.8]) (Table 1).

Diagnostic confidence (Q2) was rated on a 1–5 Likert scale. Median confidence was 5 (IQR: 4–5) across all three CT modalities for all readers combined. CTfull received the highest mean confidence rating (4.61 ± 0.63), followed by CT10dld (4.45 ± 0.71) and CT5dld (4.41 ± 0.72). Stratified by reader specialty, MSK radiologists rated confidence lower than ED radiologists across all modalities (Table 2).

Diagnostic sufficiency (Q3) was high across all CT modalities. CTfull was rated as sufficient in 91.7% of reads (110/120 [95% CI: 85.3–95.4%]), compared to 87.5% for CT10dld (105/120 [80.4–92.3%]) and 88.3% for CT5dld (106/120 [81.4–92.9%]) (Table 3).

Perceived image quality (Q4) was assessed on a 1–5 Likert scale (1 = nondiagnostic; 5 = excellent). CTfull was rated highest overall (median 4, IQR 4–5; mean 4.21 ± 0.83), followed by CT10dld (median 3, IQR 3–4; mean 3.47 ± 0.97) and CT5dld (median 3, IQR 3–4; mean 3.23 ± 0.98). CTfull was rated higher in image quality than both CT10dld and CT5dld across all case difficulty strata (easy, normal, and hard) when compared with mean values. CT10dld was rated higher than CT5dld only in the normal stratum. These results are shown in Table 4.

Inter-rater agreement on diagnostic accuracy (Q1) was assessed using Cohen’s kappa stratified by CT modality (Table 5). MSK radiologists demonstrated the highest agreement with each other across all modalities, reaching substantial agreement for CT10dld (κ = 0.760) and CT5dld (κ = 0.672), and moderate agreement for CTfull (κ = 0.429). ED radiologist agreement was Fair across all modalities (CTfull κ = 0.286; CT10dld κ = 0.351; CT5dld κ = 0.333). Cross-specialty ED–MSK pairs ranged from slight to moderate agreement, with ED2 demonstrating consistently lower agreement with MSK readers compared to ED1 across all modalities (Table 5). This notably low ED2–MSK1 agreement on CTfull (κ = 0.127) suggests reader-pair-specific variability at full dose that should be characterized before inter-rater agreement patterns on denoised low-dose images are interpreted as attributable to dose reduction itself.

Lastly, radiation dose for CT5dld (mean = 0.998 mSv) and CT10dld (mean = 1.997 mSv) was substantially lower than CTfull (mean = 19.97 mSv). These results are shown in Figure S2.

## 4. Discussion and Conclusions

Our pilot study demonstrates that simulated ULD-CT, enhanced with a DLD algorithm, achieves diagnostic accuracy and sufficiency similar to full-dose CT for the detection of acute pelvic and hip fractures across all reads and within most difficulty strata, based on descriptive comparison of point estimates and confidence intervals. We did not perform formal hypothesis testing given the clustered, multi-reader multi-case structure of the data and the limited sample size; these findings should therefore be interpreted as hypothesis-generating observations rather than confirmed statistical equivalence.

From our results, CT10dld and CT5dld demonstrated diagnostic accuracy comparable to CTfull across all reads (78.3% and 77.5% vs. 80.8%, respectively). In easy and normal cases, we found that all three CT modalities performed at uniformly high levels (87.5–92.% for easy cases, and 97.5% for normal cases). The same, however, is not true for hard cases where accuracy falls compared to easy and normal cases. It is known that denoising carries a known risk: aggressive noise suppression by CNN-based models can reduce spatial resolution and blur fine, low-contrast detail, which could plausibly reduce diagnostic performance for subtle findings. We did not perform a dedicated spatial-resolution or modulation-transfer-function analysis of the denoised images in this study, but we raise this as a plausible contributor to the lower accuracy observed in our “hard” case stratum.

Our results extend prior work demonstrating the superiority of ULD-CT over XR, suggesting that the addition of a DLD algorithm can preserve this diagnostic advantage while further reducing radiation dose to levels approaching those of standard radiography. In trauma cases, several other studies have found higher diagnostic performance of ULD-CT compared to XR [29,30]. This finding has even been shown to be consistent in non-traumatic cases as well, with ULD-CT outperforming chest radiographs in non-traumatic emergency department patients, at 20.1% detection rate with ULD-CT in comparison to 9.1% by chest radiographs [31]. However, our study incorporates a deep learning-powered denoising algorithm to further enhance the image quality of ULD-CT, potentially allowing for even greater reductions in radiation dose while maintaining diagnostic accuracy. Our findings aimed to show that ULD-CT, when combined with denoising algorithms, may one day be further explored as a potential alternative to full-dose CT in routine clinical practice, especially for “easy” and “normal” fracture patterns. However, we believe full-dose CT remains the preferred modality for “hard” cases, offering higher sensitivity and specificity as reported in several other studies [32,33].

Across all CT modalities, radiologists reported high diagnostic confidence, with median ratings of 5 (IQR 4–5) for all three modalities. Notably, the confidence advantage for CTfull was more pronounced among MSK radiologists, who rated all modalities lower than ED radiologists overall, suggesting that subspecialty expertise may heighten sensitivity to image quality differences between modalities. Taken together, these findings suggest that while CTfull remains the modality of greatest diagnostic confidence, confidence ratings for the denoised low-dose CT modalities remained high in absolute terms, which may be worth further investigation in scenarios where minimizing radiation exposure is a priority.

All CT modalities demonstrated high diagnostic sufficiency, with CTfull rated as sufficient in 91.7% of reads, compared to 88.3% for CT5dld and 87.5% for CT10dld. However, it is important to note that this assessment is subjective and is therefore a notable limitation. The practical implication of this finding is notable: even at 5% of standard radiation dose, readers indicated on the study questionnaire that the imaging was sufficient to render a diagnosis in most cases. However, this in-study sufficiency rating is not equivalent to a real-world decision to forgo additional imaging and should not be interpreted as such. These findings suggest that DLD-enhanced ULD-CT warrants further prospective investigation as a diagnostic tool in the acute trauma setting, rather than supporting its use as a standalone or first-line modality at this stage.

Imaging quality analyses revealed that CTfull provided the best quality overall, with CT10dld and CT5dld rated lower across all difficulty strata. Importantly, however, this reduction in perceived image quality did not translate into corresponding losses in diagnostic accuracy or sufficiency, suggesting that combined pipeline (simulated image + denoised algorithm applied) preserved sufficient diagnostic information despite lower subjective quality ratings. This finding aligns with a recent meta-analysis indicating that ULD-CT can achieve diagnostic accuracy (97.6% sensitivity and 99.6% specificity) comparable to CTfull (99.4% sensitivity and 99.1% specificity), while reducing radiation exposure by up to 87–99% [34]. Taken together, accuracy and sufficiency remained similar between DLD-enhanced ULD-CT and CTfull despite measurably lower image quality ratings, a pattern that warrants further future investigation.

Here, we observed that inter-rater agreement was highest among MSK radiologists, reaching substantial agreement on CT10dld and CT5dld compared to fair agreement among ED radiologists across all modalities. ED2 demonstrated consistently lower agreement with all other readers regardless of modality, though its inclusion reflects real-world variability in radiologist experience and underscores the importance of subspecialty training. However, these results were based on only 30 cases, and as such, we were unable to statistically compare them across modalities. Lastly, radiation doses for CT5dld (mean 0.998 mSv) and CT10dld (mean 1.997 mSv) were substantially lower than CTfull (mean 19.97 mSv), consistent with the intended dose reduction in the simulated protocols.

Our pilot study, however, is not without limitations. First, our study simulated ultra-low-dose CT images rather than prospectively acquiring ULD-CT scans due to practical constraints, as modifying CT scan protocols in the clinical care setting for research purposes would be challenging. Prior work has demonstrated that simulated low-dose CT closely approximates the image quality and noise texture of prospectively acquired low-dose scans, supporting the validity of this approach [35,36]. Nevertheless, we acknowledge that true patient-based low-dose acquisitions may exhibit additional variability not fully captured by simulation. However, this research can help support the implementation of ULD-CT in the clinical setting through quality improvement initiatives. Second, our sample size was limited to 30 patients with exclusion of patients with marked demineralization, hardware, and/or large body habitus, which may influence the generalizability of our findings. This smaller sample size may have contributed to the wider confidence intervals observed in our data. Classification of the fractures as “easy” or “hard” is subjective and may also serve as a limitation of this study. There is no official or objective standard for determining “easy” versus “hard” pelvic and hip fractures in the literature, so we established a two-person agreement on the fracture difficulty to provide some standardization and excluded cases where there would be clear limitations in the imaging (high degrees of osseous demineralization, artifacts due to hardware, large body habitus). Third, while our study provides promising preliminary results supporting the use of ULD-CT, the broader generalization of full-dose or low-dose CT requires further investigation. Additionally, although radiographs were required as part of the inclusion criteria, they were not included as a direct comparator in the reader study, and clinical history was intentionally withheld from readers to minimize interpretive bias. As a result, this pilot cannot speak to how DLD-enhanced ULD-CT would perform relative to radiography, or with the benefit of clinical context, as would occur in routine practice. Building on this pilot, we recommend that future work: (1) prospectively acquire true low-dose CT rather than relying solely on simulation; (2) recruit a larger, multi-institutional cohort with a prevalence-representative (non-curated) case mix, or explicitly model the enrichment if a curated design is retained; (3) include a sim-only (pre-denoising) reader arm to isolate the incremental contribution of the denoising algorithm; (4) prespecify a clinically justified noninferiority margin and perform a corresponding power calculation before testing an equivalence/noninferiority hypothesis; and (5) report sensitivity, specificity, and reader-level performance rather than aggregate accuracy alone.

In conclusion, in this descriptive pilot study, DLD-enhanced ULD-CT showed accuracy and sufficiency similar to full-dose CT for the detection of acute pelvic and hip fractures, at a fraction of the radiation dose, though full-dose CT was rated higher in perceived image quality and confidence and outperformed denoised low-dose CT numerically in hard cases. These findings are hypothesis-generating rather than definitive, and support further prospective investigation of denoised low-dose CT as a potential option for straightforward presentations where radiation minimization is a priority, rather than justifying a change in current clinical practice. Larger, prospective studies with true low-dose acquisitions, a broader range of patients, formal statistical modeling of the multi-reader multi-case structure, and radiography as a direct comparator are needed before broader clinical adoption can be considered.

## Figures and Tables

**Table 1:** Diagnostic accuracy by CT modality and case difficulty. CI of 95% computed using Wilson score interval. Note: 95% CIs computed via Wilson score interval treating the 120 reader-reads as independent observations. CTfull = standard full-dose CT; CT10dld = 10% dose denoised; CT5dld = 5% dose denoised.

Stratum	Modality	Correct/Total	Accuracy (%)	95% CI
**Overall Cases**				
	CTfull	97/120	80.8%	[72.9, 86.9]
	CT10dld	94/120	78.3%	[70.1, 84.8]
	CT5dld	93/120	77.5%	[69.2, 84.1]
**Easy Cases**				
	CTfull	35/40	87.5%	[73.9, 94.5]
	CT10dld	37/40	92.5%	[80.1, 97.4]
	CT5dld	37/40	92.5%	[80.1, 97.4]
**Normal Cases**				
	CTfull	39/40	97.5%	[87.1, 99.6]
	CT10dld	39/40	97.5%	[87.1, 99.6]
	CT5dld	39/40	97.5%	[87.1, 99.6]
**Hard Cases**				
	CTfull	23/40	57.5%	[42.2, 71.5]
	CT10dld	18/40	45.0%	[30.7, 60.2]
	CT5dld	17/40	42.5%	[28.5, 57.8]

**Table 2:** Diagnostic confidence (Q2) by CT modality and reader specialty. Confidence rated on a 1–5 Likert scale (1 = not at all confident, 5 = extremely confident).

Modality	Reader	Median (IQR)	Mean ± SD
**All Readers**			
CTfull	All	5 (4–5)	4.61 ± 0.63
CT10dld	All	5 (4–5)	4.45 ± 0.71
CT5dld	All	5 (4–5)	4.41 ± 0.72
**ED Radiologists**			
CTfull	ED	5 (5–5)	4.91 ± 0.28
CT10dld	ED	5 (5–5)	4.80 ± 0.46
CT5dld	ED	5 (5–5)	4.77 ± 0.49
**MSK Radiologists**			
CTfull	MSK	4 (4–5)	4.30 ± 0.73
CT10dld	MSK	4 (4–4)	4.10 ± 0.74
CT5dld	MSK	4 (4–4)	4.05 ± 0.80

**Table 3:** Diagnostic sufficiency (Q3) by CT modality. Sufficiency defined as radiologist response of “Yes” to whether imaging was sufficient to make a diagnosis.

Modality	Sufficient	Rate (%)	95% CI
CTfull	110/120	91.7%	[85.3, 95.4]
CT10dld	105/120	87.5%	[80.4, 92.3]
CT5dld	106/120	88.3%	[81.4, 92.9]

**Table 4:** Perceived image quality (Q4) by CT modality and case difficulty. Image quality rated 1–5 (1 = nondiagnostic, 5 = excellent).

Stratum	Modality	Median (IQR)	Mean ± SD
**Overall Cases**			
	CTfull	4 (4–5)	4.21 ± 0.83
	CT10dld	3 (3–4)	3.47 ± 0.97
	CT5dld	3 (3–4)	3.23 ± 0.98
**Easy Cases**			
	CTfull	4 (4–5)	4.17 ± 0.87
	CT10dld	3 (3–4)	3.27 ± 0.96
	CT5dld	3 (2–4)	3.15 ± 0.98
**Normal Cases**			
	CTfull	4 (3–5)	4.17 ± 0.84
	CT10dld	4 (3–4)	3.60 ± 0.96
	CT5dld	3 (3–3)	3.15 ± 0.876
**Hard Cases**			
	CTfull	4 (4–5)	4.28 ± 0.78
	CT10dld	3 (3–4)	3.52 ± 0.99
	CT5dld	3 (3–4)	3.40 ± 0.82

**Table 5:** Inter-rater agreement (Cohen’s κ) by CT modality. Cohen’s kappa (κ) interpretation: <0.20 Slight, 0.21–0.40 Fair, 0.41–0.60 Moderate, 0.61–0.80 Substantial, >0.80 Almost Perfect. C.I = Confidence Interval.

Modality	Reader Pair	Cohen’s κ [95% C.I]	Interpretation	Specialty Pair
**CTfull**				
	ED1 vs. ED2	0.286 [0.0, 0.571]	Fair	ED–ED
	ED1 vs. MSK1	0.259 [0.0, 0.783]	Fair	ED–MSK
	ED1 vs. MSK2	0.429 [0.0, 0.839]	Moderate	ED–MSK
	ED2 vs. MSK1	0.127 [0.0, 0.420]	Slight	ED–MSK
	ED2 vs. MSK2	0.462 [0.156, 0.757]	Moderate	ED–MSK
	MSK1 vs. MSK2	0.429 [0.0, 0.870]	Moderate	MSK–MSK
**CT10dld**				
	ED1 vs. ED2	0.351 [0.0, 0.672]	Fair	ED–ED
	ED1 vs. MSK1	0.520 [0.0, 0.889]	Moderate	ED–MSK
	ED1 vs. MSK2	0.520 [0.0, 0.889]	Moderate	ED–MSK
	ED2 vs. MSK1	0.514 [0.194, 0.889]	Moderate	ED–MSK
	ED2 vs. MSK2	0.351 [0.0, 0.672]	Fair	ED–MSK
	MSK1 vs. MSK2	0.760 [0.286, 1.00]	Substantial	MSK–MSK
**CT5dld**				
	ED1 vs. ED2	0.333 [0.0, 0.667]	Fair	ED–ED
	ED1 vs. MSK1	0.524 [0.0, 0.870]	Moderate	ED–MSK
	ED1 vs. MSK2	0.510 [0.07, 0.870]	Moderate	ED–MSK
	ED2 vs. MSK1	0.294 [0.0, 0.630]	Fair	ED–MSK
	ED2 vs. MSK2	0.432 [0.067, 0.769]	Moderate	ED–MSK
	MSK1 vs. MSK2	0.672 [0.242, 1.00]	Substantial	MSK–MSK

## Data Availability

The datasets used and/or analyzed during the current study are available from the corresponding author on reasonable request.

## Supplementary Materials

The following supporting information can be downloaded at: https://www.mdpi.com/article/10.3390/diagnostics16172862/s1, Figure S1. Survey questions for attending radiologists to answer following review of image. Radiological images were randomized and each attending radiologist completed the following survey after independently reviewing the images for each case. Figure S2. Radiation dose across different imaging modalities (CTfull, CT5dld, and CT10dld, *n* = 30 patients for each group). Data shown as a logarithmic scale for easier visualization. Box plots display the median (center line), interquartile range (box), and range excluding outliers (whiskers), with outliers plotted individually.

## Abbreviations

BMI	Body Mass Index
CI	Confidence Interval
CNN	Convolutional Neural Network
CT	Computed Tomography
CT5sim	Computed Tomography with 5% radiation dose
CT5dld	Computed Tomography with 5% radiation dose, denoised
CT10sim	Computed Tomography with 10% radiation dose
CT10dld	Computed Tomography with 10% radiation dose, denoised
CTdld	Computed Tomography denoised low dose
CTfull	Full-dose Computed Tomography
DICOM	Digital Imaging and Communications in Medicine
DLD	Deep Learning-based Denoising
DLR	Deep Learning Reconstruction
ED	Emergency Department
IRB	Institutional Review Board
MSK	Musculoskeletal
mSv	Millisievert (unit of radiation dose)
PACS	Picture Archiving and Communication System
PGY	Postgraduate Year (Residency Level)
ULD-CT	Ultra-Low Dose Computed Tomography
XR	X-ray (Radiograph)
