# Supplementary material for: Feasibility of AI-Denoised Ultra-Low-Dose CT for Detection of Acute Pelvic and Hip Fractures: A Pilot Multi-Reader Study
Source: Diagnostics (Basel). 2026 Sep 6;16(17):2862. doi: 10.3390/diagnostics16172862 (PMC13564755; doi:10.3390/diagnostics16172862)

Figure S1. Survey questions for attending radiologists to answer following review of image. Radiological images were randomized and each attending radiologist completed the following survey after independently reviewing the images for each case.

1. What is the diagnosis?
    - Please give an impression as you would in a formal radiology report (including all relevant findings and descriptions).
    - If there are no findings, please write "normal".

2. How confident are you in your final diagnosis?
    - Please answer on a scale of 1-5.

1	2	3	4	5
Not at all confident	Moderately not confident (unsure)	Neither confident nor not confident (neutral)	Moderately confident	Extremely confident

3. Was the provided imaging sufficient to make the diagnosis? (Yes or no). If not, which imaging modality would you recommend/ask for?
    - Please answer as: "Yes" or "No, (please *specify imaging modality requested*)"

4. How was the diagnostic imaging quality?
    - Please answer on a scale of 1-5.

1	2	3	4	5
Nondiagnostic	Suboptimal with major technical limitations	Some minor technical limitations	Expected/average quality	Excellent quality without technical limitations

Figure S2. Radiation dose across different imaging modalities (CTfull, CT5dld, and CT10dld, n = 30 patients for each group). Data shown as a logarithmic scale for easier visualization. Box plots display the median (center line), interquartile range (box), and range excluding outliers (whiskers), with outliers plotted individually.

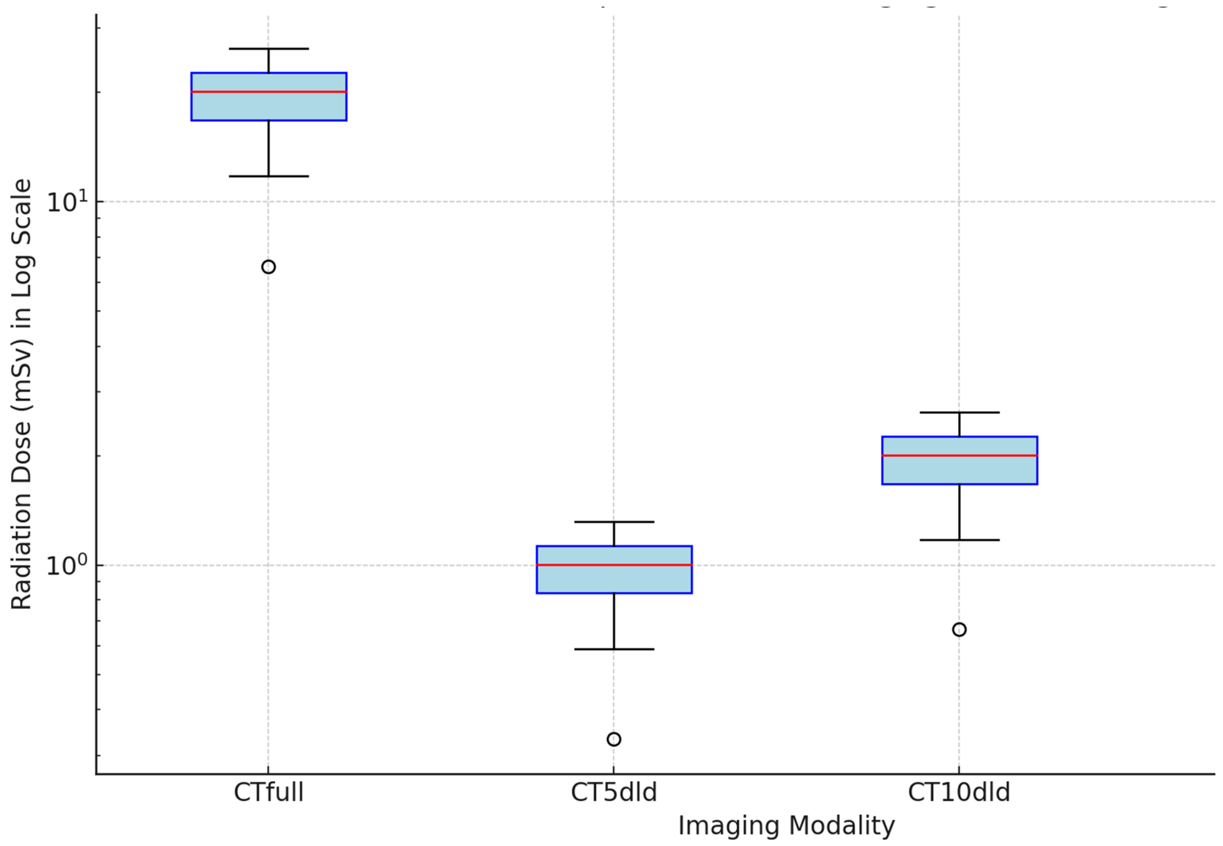

Supplement: Supplementary file 1 [file diagnostics-16-02862-s001.zip › diagnostics-4365541-supplementary.pdf]
